# A literature review on prosthetically designed guided implant placement and the factors influencing dental implant success

**DOI:** 10.1038/s41415-024-7050-3

**Published:** 2024-02-09

**Authors:** Adam Nulty

**Affiliations:** International Digital Dental Academy, 128 Harley Street, London, W1G 7JT, UK; PhD Student, University of Leeds, Woodhouse Lane, Leeds, LS2 9JT, UK

## Abstract

This is an extensive review of the literature on prosthetically designed implant planning with particular regard to the factors influencing dental implant success. Electronic searches on PubMed and the Cochrane Central Register of Controlled Trials and manual searches were performed. The author selected the studies according to the inclusion and exclusion criteria. Meta-analysis of implant placement accuracy and a qualitative review of potential influencing factors were performed.

This literature review will explore prosthetically designed implant placement and its influence on dental implant success. By examining the factors that impact implant outcomes, this review aims to provide clinicians with a comprehensive understanding of the key considerations and best practices in achieving successful implant placements. Ultimately, this knowledge can contribute to enhancing patient care and the long-term success of dental implant treatments.

## Key points

- This paper underscores the importance of prosthetically guided implant placement, which ensures that the implant is placed in a position that is optimal for the final prosthetic result. This approach not only enhances aesthetic outcomes but also ensures functional efficacy, contributing to the longevity and success of the implant.
- The findings highlight various factors that influence the success of dental implants, such as the quality and quantity of the bone where the implant is to be placed, the patient's oral hygiene and the surgeon's skills and expertise. Understanding these factors allows dental professionals to optimise pre-surgical planning and post-surgical care to maximise implant success rates.
- By focusing on prosthetically designed implant placement and understanding the factors that influence implant success, dental professionals can enhance patient outcomes. This approach minimises the risk of implant failure, ensures optimal functional and aesthetic results and can potentially reduce the need for additional procedures, thereby improving patient satisfaction and overall experience.

## Introduction

The field of dental implantology has witnessed remarkable advancements in recent years, revolutionising the way missing teeth are replaced. Dental implants provide a reliable and long-lasting solution for individuals seeking to restore their oral function and enhance their aesthetic appearance. However, achieving successful outcomes in dental implant treatment requires a comprehensive understanding of various factors influencing implant success.

In dental implant treatment, guided surgery allows the clinician to think forward and provide a prosthetically designed implant placement that considers the future prosthesis virtual envelope to plan the position and depth of the implant. This is important to create a more aesthetic outcome with an appropriate and cleanable emergence profile.^1^^,^^2^^,^^3^^,^^4^

An inappropriate position of the implant can lead to future problems that may be simple, such as difficulty in cleaning, or more impactful on long-term success, such as peri-implantitis.^5^^,^^6^^,^^7^^,^^8^

Due to these benefits, surgical guides have increased as clinicians look to increase predictability and accuracy over freehand surgery.^9^^,^^10^^,^^11^

This literature review explores the critical aspects of prosthetically designed implant placement and the factors that play a crucial role in determining the success of dental implant procedures. By examining and synthesising a wide range of published studies, this review provides a comprehensive overview of the current knowledge in this field, highlighting the critical considerations for optimal implant placement.

Prosthetically designed implant placement involves a meticulous approach that considers the final prosthetic outcome during the planning and execution phases of dental implant treatment. This approach considers the aesthetic and functional requirements of the patient, ensuring that the implant placement aligns with the desired final result. By emphasising prosthetic-driven implant planning, clinicians can harmonise the implant-supported prosthesis and the natural dentition, improving patient satisfaction and treatment success.

Furthermore, this literature review will explore various factors influencing dental implant success. Factors such as bone quality and quantity, implant stability, occlusal forces, surgical technique and patient-related factors will be examined in detail. Understanding these factors and their impact on implant outcomes is paramount for clinicians, as it helps guide treatment planning and decision-making processes, leading to more predictable and successful results.

The implications of this literature review extend beyond the clinical realm. This review aims to contribute to the existing body of knowledge by synthesising existing evidence, providing valuable insights for researchers and practitioners alike. Additionally, the findings of this review help shape future research directions and clinical guidelines, ultimately improving the overall quality of dental implant treatments.

## Materials and methods

### Search strategy

A thorough exploration of electronic literature was performed, confining the search to studies in the English language published from 2008 through to March 2023, utilising the PubMed database as the resource. The inclusion criteria embraced a range of study designs, encompassing randomised controlled trials (RCTs) as well as prospective and retrospective clinical cohort studies. The electronic search through the databases was strategically guided by the following search terms: (dental implantation or dental implant) in conjunction with (computer-assisted surgery or computer-aided design or computer-aided surgery or computer-guided surgery or digital dentistry or guided implant surgery) and also involving (dimensional measurement accuracy or dental implant deviation or dental implant accuracy or dental implant precision).

### Literature found

A total of 66 papers containing information relating to one of the search terms were identified. These papers were screened and many clinical studies were identified, relating to either studies of just virtual implant planning accuracy or studies comparing the accuracy of different types of implant guides when measured against their 3D-planned counterparts. The literature review is therefore based on retrospective and prospective cohort studies that were gathered from the search.

Once articles had been identified, they were analysed according to the methodology used, the risk of bias, the interpretation of the findings and the conclusions drawn. Further inclusion criteria were implemented to focus the number of studies to be reviewed by selecting studies more comparable to the abovementioned objectives. References to information such as the accuracy of 3D printers and the history of implants were included.

### Reasons for study exclusion

The reasons some search results were excluded from the literature review were:

- Results based on older technologies that had now been superseded with newer, more accurate technologies
- Data affecting the discussion or results that were unrelated to the search terms.

There were a low number of studies based on guided implant placement in general and an even lower number of studies related to edentulous guided surgery, whether flapless or not, hence the low number of search results gathered.

### Data obtained

The primary data obtained were relevant to the accuracy of the different ways of creating a digital impression. There were a number of articles included in the literature review that concerned measuring or reflecting on other studies that measured the accuracy of the various types of implant drill guides currently on the market.

## Literature results

Hultin *et al.* discussed that conventional manual manufacturing of guides is complex and labour-intensive and that manual errors are possible.^10^

Computer-designed guided surgery is centralised, fully automated and accurate. However, the costs are higher and may be slower if shipping is involved.

Hutling and Columbo^10^^,^^12^ both discussed reported complications in the retrospective results:

- Fracture of splints
- Misplacement due to misfabrication of guide
- Lack of primary stability
- Insertion of wider implants than planned
- Limited oral aperture restricting usage of tools
- Misfit of guides
- Uncontrolled gingival removal.

The results given in literature related to guided surgery can be summarised according to the steps in which guided implant surgery is traditionally carried out and the stages involved:

- Data matching and virtual planning
- Guide design and manufacture
- Guided surgery components
- Guided surgery drilling and implant placement
- Human error.

After the advent of dental implants, their use has become more prevalent, and so have implant-related complications and sequela. Improper design, planning and surgical placement without adhering to the principles discussed above and placement not taking into account the final prosthetic design has led to an increased demand for ways to minimise these errors. To try and overcome these potential problems, over time, as technology has improved, surgical guides have become more prevalent. Implantologists have embraced recent technological advancements, which also present an increased number of patients potentially exposed to the complications of limitations in the accuracy of current guided surgery. These guides have developed from simple analogue, lab-made prosthetic envelopes to fully guided, digitally designed and produced guides that aim to increase accuracy, better surgical execution and prevent iatrogenic damage.

## Data matching and virtual planning

When we plan the implant surgery in a 3D programme, the eventually produced template is designed on the 3D impression (an STL file produced in the first stage). This is, however, just the surface of the oral environment. The 3D cone-beam computed tomography (CBCT) scan is a separate image and the two datasets are then combined to provide a whole representation of the patient that can be used for guided surgery implant planning. Referencing the 3D impression data to the CBCT involves manual or computer-aided matching of common elements that can be seen on both 3D image sets.

Data matching has been evolving and the most recent software algorithms used in the main computer-aided guide design software packages have an automatic calibration and matching function. This is optional in some cases.

As discussed by Behneke *et al.*,^13^ if the impression data are referenced poorly but printed accurately, it will fit well but will guide the drills and the implant placement in the wrong position. Accurate referencing and data matching are, therefore, critically important.^13^

## Guide design and manufacture

### Types of drill guide to be produced

One of the significant factors affecting the accuracy of guided implant surgery is the type of guide being designed and used, be it dentate, edentulous, that is, mucosa-borne, or bone-supported. Different types of implant drill guides may be produced when considering the simultaneous placement of several implants in the edentulous arch:

- Dentate-borne
- Mucosa-borne
- Bone-supported.

**Fig. 1:**
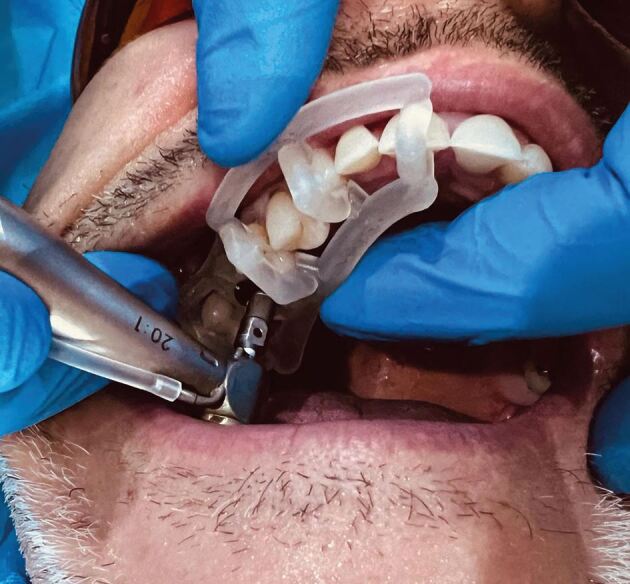
Dentate-guided implant surgery

The accuracy variation between these guides is the guide's stability on the soft tissues, teeth and bone (Fig. 1).

Fixation pins can be used to secure the latter two types in place and the position and depth of these fixation pins are designed within the drill guide design software.

The edentulous mucosa-borne drill guides can be used with or without a crestal incision to expose the buccal bone surface, whereas obviously, the bone-supported drill guide requires full exposure of the bone with large incisions and full flaps reflected. Previous studies have suggested that the movement during drilling when using a surgical guide can affect the accuracy of the creation of the osteotomy.^14^^,^^15^Fixation pins, therefore, aim to reduce this error by maintaining the position of the guide.^16^^,^^17^

With edentulous-guided surgery, the resulting accuracy is due to problems referencing the digital impression and the CBCT scan. Surgical complications result from deviations and misplacement due to the edentulous guide's fit and/or use as the mucosa compresses or rotates.^3^

The initial theory suggested that as the mucosa is not a fixed platform, the bone-supported drill guides would be more accurate. However, current thinking^18^ suggests this is not the case, as it is difficult to expose the bone surface in a way that replicates the digital version of the bone surface used to create the drill guide due to both the fact that not all of the soft tissue can be removed entirely on flap creation and also the accuracy of the bone surface extraction algorithm that chooses where the boundary of the bone is.^18^

Dentate guides are inherently less affected by these issues as they sit on teeth. The teeth present also bridge the bone and soft tissue and thus appear on both the CBCT and impression data, meaning reference points are more expansive.

As clinicians and users of these implant drill guides, we must therefore understand the causes of any inaccuracy to avoid these effects in practice.

### Accuracy of CBCT scanners on digitisation of models

Becker *et al.*^19^studied the accuracy of five of the most common light-based model 3D scanners and eight of the most common CBCT scanners that are able to digitise models into a 3D STL. The study involved the digitisation of ten patient models. The digitised models and their corresponding surfaces were compared using mesh lab v1.3.4. A statistical analysis using box plots, a paired t-test and a Friedman test were used to compare with a significance level of 5%. The light-based 3D scanners had a minor mean deviation in accuracy which was ± SD: 0.017-0.004 mm. The most accurate CBCT scanner in the studies was the Carestream 8100 3D CBCT scanner which had a mean deviation of ± SD 0.03. Therefore, it concluded that light-based scanners are more accurate where the paired t-test revealed an average difference in measured distance between light and CBCT scanners as 0.046 mm.

However, colour heat maps of the light-based scanners still showed regions in the palate of up to 1 mm deviation. When we are looking at guided surgery studies, such as this research study, this is more clinically relevant, as these areas of poor fit from a poor scan will cause the overall guide to sit improperly and result in implants placed in the wrong position. Another point of discussion is that the Becker paper used dentate orthodontic models, which would result in more favourable alignment in guided implant surgery software.^19^

### CBCT and digital model data matching

**Fig. 2:**
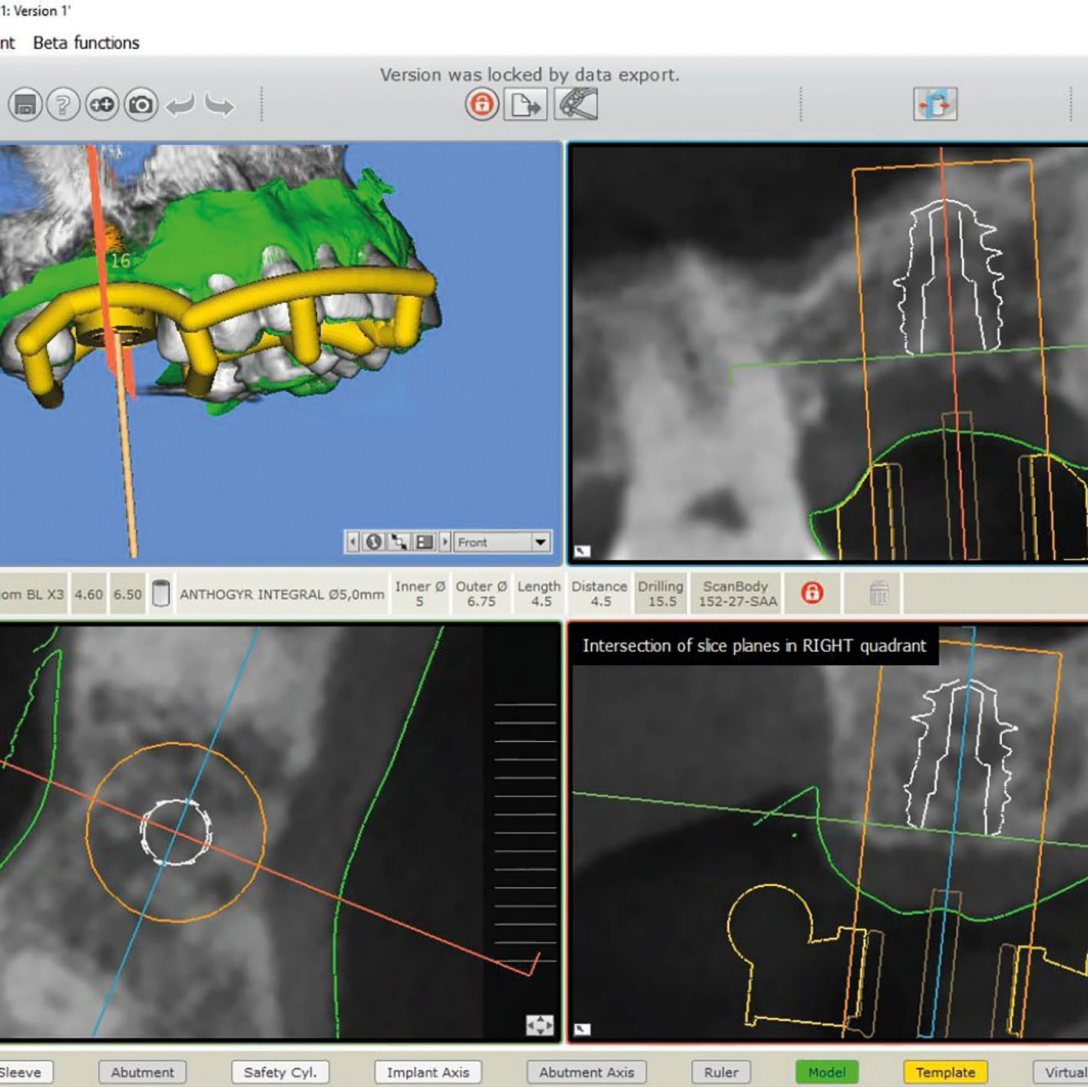
STL matching with SMOP guide software

As discussed above, referencing the 3D impression data to the CBCT involves either manual or computer-aided matching of common elements that can be seen on both 3D image sets (Fig. 2). Historically, this was done manually, with transfer devices such as the X1-table, but with the evolution of digital software, this is becoming more commonly autonomous.

**Fig. 3:**
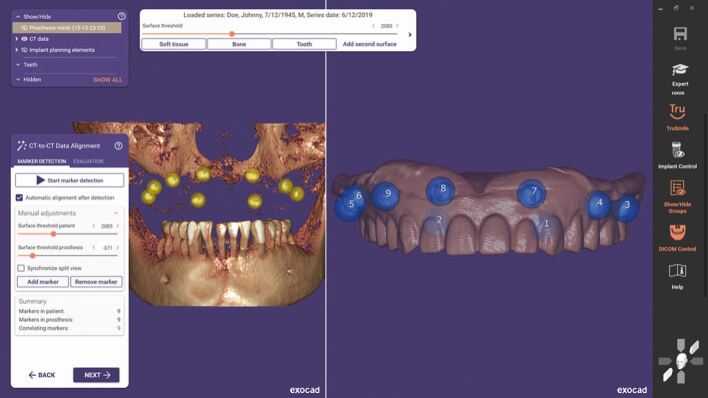
CBCT to CBCT dual scan data matching with Exoplan software

Kernen *et al.*^20^ aimed to compare manual, lab-based matching with the fabrication of manual transfer devices with digitally designed printed templates. The mean lab-based matching deviated by 0.31/0.32 mm in the two horizontal planes and 0.5 mm apically. The digitally planned templates produced a mean deviation of 0.16-0.23 mm horizontally and 0.25 mm apically. Kernen concluded that templates that had a surface scan matched with CBCT data were statistically significantly more accurate than laboratory-fabricated templates: ‘within the limits of the study, it can be concluded that a higher accuracy may be achieved if templates are virtually designed and printed after superimposing a surface scan with a CBCT in order to transform the virtual plan into reality, applying the SMOP technology' (Fig. 3).^20^

Data matching has been evolving and the most recent software algorithms used in the main computer-aided guide design software packages have an automatic calibration and matching function. This is optional in some cases.

### Accuracy of the 3D printer

3D printers are of various types. The most common type of 3D printer used in medicine and dentistry are stereolithography (SLA) or digital light processing printers, which use a laser or a light projector to cure the resin in a chamber as the mount raises. There are two types of accuracy in the printing of models and guides with 3D printers of this type: the Z-plane accuracy and X/Y-plane accuracy.

All 3D prints also require supports to be virtually added to the 3D STL of the designed guide so that the object prints accurately with no distortion. Proper orientation and placement in the print software are therefore crucial so that the fitting surface has no supports in contact with the fitting surface or where the guide components, such as keys, spoons or drills, fit.

The X/Y-plane accuracy is the most crucial as this depends on the spot size of the laser.^21^ The laser of the Form 2 SLA printer and the Form 3 SLA printer (commonly used 3D printers in dental surgery due to the biocompatible surgical guide SLA resin being commonly available and constant developments of other resins for dentures, models etc) is 140 microns and 85 microns, respectively, and the accuracy to within a 95% confidence is around 10 microns above or below this. More expensive 3D lab-based SLA printer accuracy can be less than this, of up to 25 microns.

The spot size of the laser, the size of the steps in between each layer and the Z-plane can be more accurate depending on the quality of the 3D printer, but even commonly available printers, such as the Form 2, have a selectable Z-plane accuracy of up to 25-50 microns. More significant Z-plane steps mean that each layer or ‘slice' is thicker and therefore the overall print time is reduced. Formlab's own study of accuracy used nine prints of different Form 2 printers measuring seven different dimensional components with the resulting standard deviation of ‘95% of prints measured to within 240 μm or less (0-24 mm) of the designed dimension'.^21^

The manufacturers of the 3D printers recommend that the guides are oriented either vertically or with up to a 45-degree angulation to benefit from less need for supports and also to utilise the extra accuracy from the Z-plane.

A recent study^22^ on the accuracy of a broad range of 3D printers showed that the current range of 3D printers could produce clinically acceptable levels of accuracy with mean accuracy ranging from -0.016 (± 0.025) to 0.149 (± 0.094). In designing surgical implant guides, more recent computer-aided design software allows you to take these errors into account by tailoring tolerance parameters to suit the model and brand of printer, which may have a smaller or larger average deviation.

### Drill guide platform

When considering the planning and placement of several implants in the edentulous arch, there are different types of implant drill guides that may be produced:

- Mucosa-borne
- Bone-supported.

**Fig. 4:**
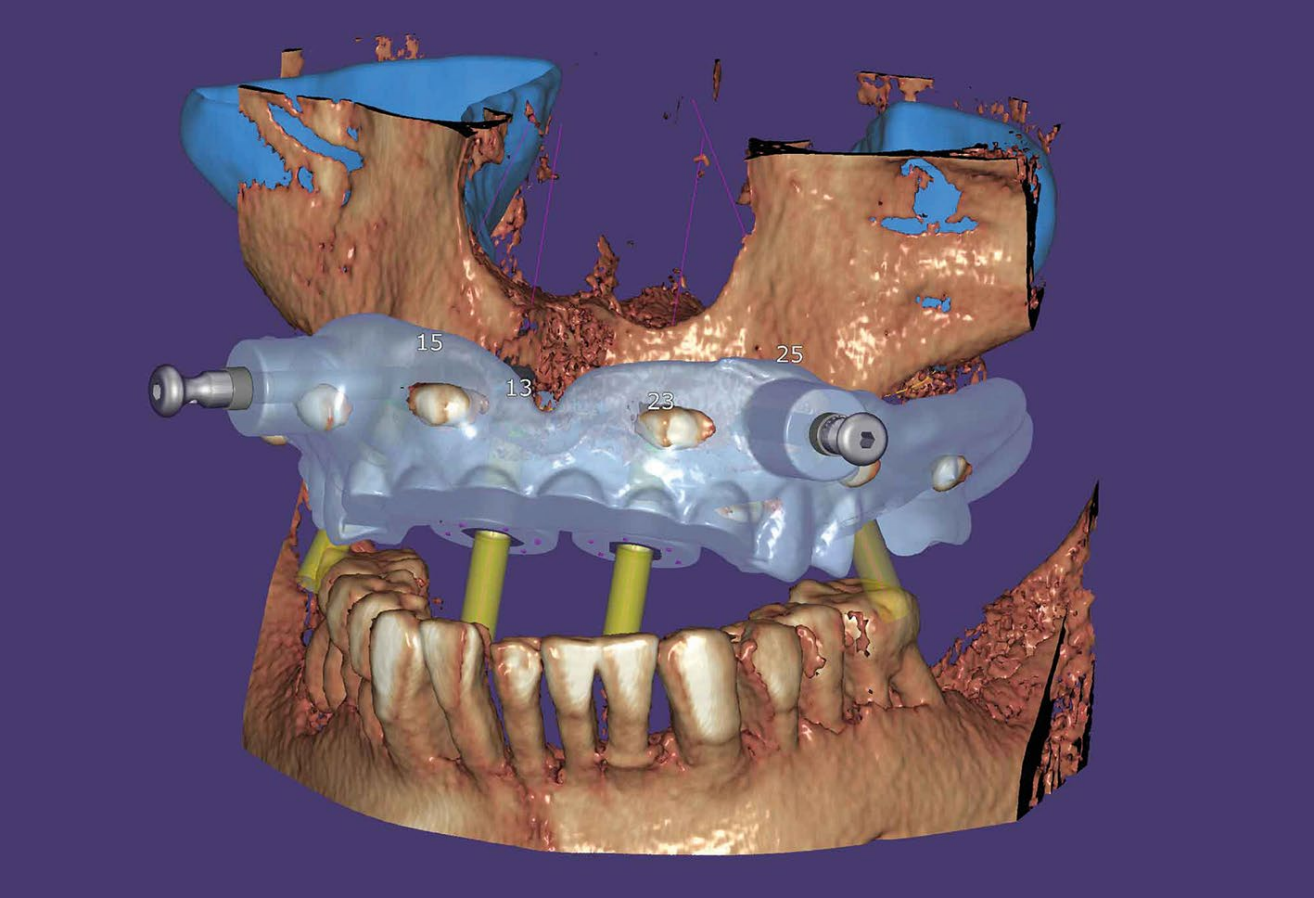
Fixation pins added buccally to the edentulous mucosa borne guide

Fixation pins are employed to stabilize both types of drill guides, with their position and depth determined using drill guide design software (see Fig. 4). Mucosa-borne drill guides can be utilized with or without a crestal incision for revealing the buccal bone surface.

 In contrast, bone-supported drill guides necessitate complete bone exposure through extensive incisions and the reflection of full flaps.

Arisan *et al*.^18^ have shown that mucosa-borne guides are more accurate in a comparative study. The study was quite a significant in vivo study, enrolling 54 patients. A total of 294 implants were planned and placed using 60 guides of varying types of edentulous stents: mucosa-borne, tooth-borne, fixed and with multiple parts. A post-operative CBCT was taken to compare the planned and the placed positions. Kruskal-Wallis and Mann-Whitney U tests were used with a p-value of 0.05. The conclusion was that ‘implants that were placed by bone-supported guides had the highest mean deviations (5.0° ± 1.66° angular, and 1.70 ± 0.52 mm and 1.99 ± 0.64 mm for implant coronal and apex, respectively), whereas the lowest deviations were measured in implants that were placed by mucosa-supported guides (2.9° ± 0.39° angular, and 0.7 ± 0.13 mm and 0.76 ± 0.15 mm for implant coronal end and apex, respectively)'.

### Edentulous guided surgery

There are various inaccuracies that occur in each stage within the planning and construction of the edentulous implant drill guide^23^ and the resulting deviation is potentially limited in terms of achieving completely accurate final positioning. Several variable factors may create errors in this process, including CBCT scans that have inherent distortion, the capture of the surface impression data, the 3D printing of the designed drill guide, mucosal compression, distortion at all stages and drill wobble due to tolerance gaps.

In a fully edentulous arch, pre-operative planning is critical for effective restorative outcomes in full-mouth rehabilitation.^12^ The use of computerised implant planning using CBCT has increased dramatically in recent years. Through this process, dental implant placement is made through the use of implant drill guides used to create the osteotomy with the final prosthetic position in mind.^24^

Advances in computer-aided design/computer-aided manufacturing (CAD/CAM) technology have made it possible to combine surgical plan digital data with prosthetic designs for immediate restoration at the time of implant placement. Through computer-guided implant placement, the clinician can effectively and accurately replace the tooth with a temporary one that is premade at the time of implant placement. In some situations, elevating the mucosal flap to assess the bone is not required, resulting in a flapless surgical operation.^25^

According to Kernen^20^and Florian *et al*., using 3D-printed templates produced from a surface scan and CBCT matching data in the virtual plan, it is feasible to achieve improved implant placement accuracy.^26^ However, this pre-placement, premade temporary, is more challenging to perform in full arch implant cases, where deviations are larger, and a full arch restoration which requires passivity in each implant connection is difficult. However, the use of an implant drill guide improves precision and minimises complications, such as damage to the mandibular nerve, sinus perforation, fenestrations and dehiscences. Furthermore, ‘prosthetically directed implant placement using computer software can ensure precise placement and predictable outcomes'.^27^

**Fig. 5:**
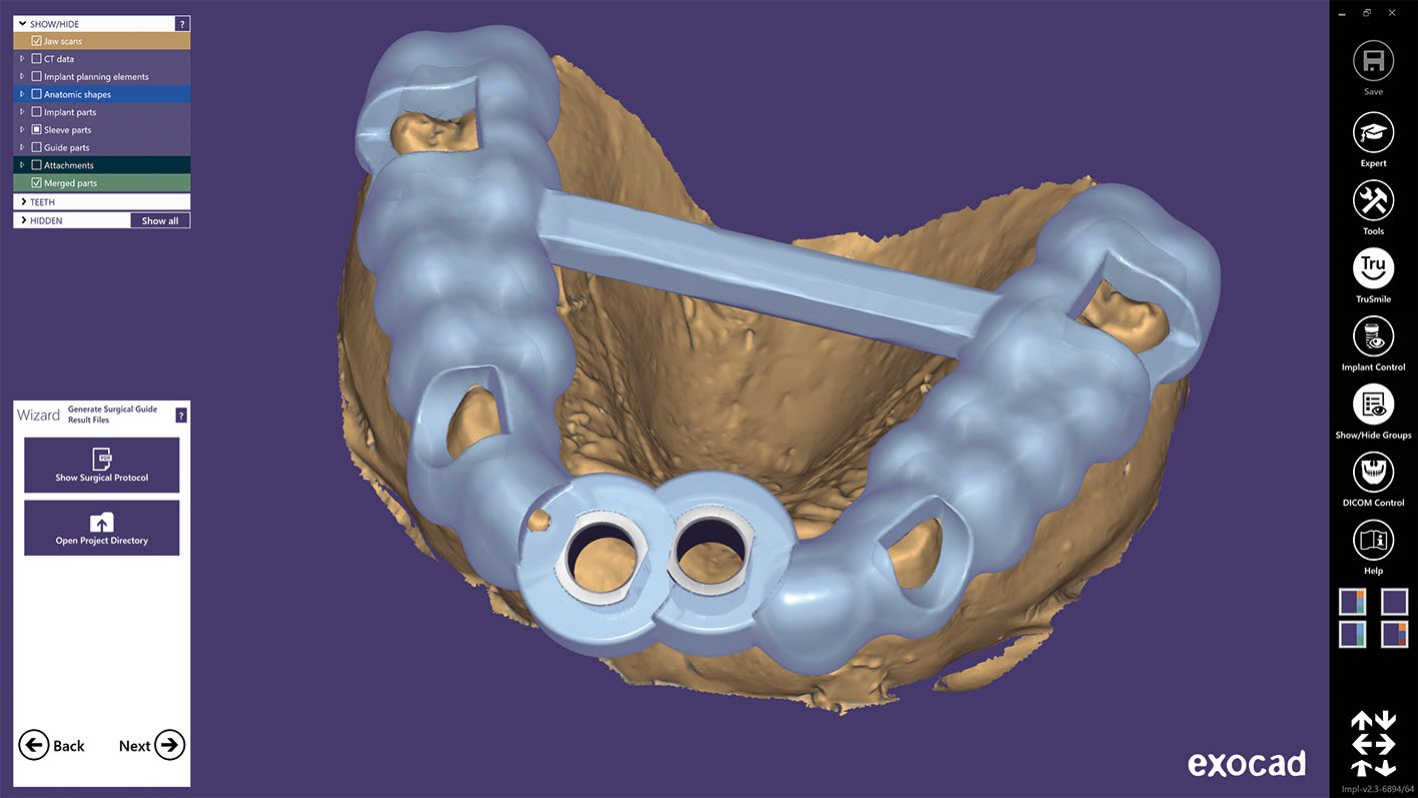
Designing the surgical guidance for implant placement with Exoplan-guided surgery software

In the creation of implant drill guides after digital planning, the discussions in this thesis have considered what errors are possible during this planning, osteotomy creation with drills, and the placement process. A full arch guide created from a virtual plan for edentulous cases has its own specific factors relating to accuracy.^28^ A phenomenon known as shrinkage has been seen, in which CBCT readings were smaller than the actual values. When the plaster model or intra-oral scanner values indicate shrinkage at the same rate as the CBCT data, the matching of digital data is considered to be trustworthy.^29^ However, if the shrinkage rate varies dramatically from one sample to the next, the digital data acquired become untrustworthy. Komuro et al.^28^ found results in their study of this shrinkage that ‘all values measured with CBCT were significantly smaller than that of a model scanner, iOS and control (p <0.001). The model scanner shrinkage was 0.37-0.39%, iOS shrinkage was 0.9-1.4% and CBCT shrinkage was 1.8-6.9%. There were statistically significant differences among the shrinkage with iOS, CBCT and model scanner (p <0.001)' (Fig. 5).^28^

## Guided surgery components

### Types of drill guide sleeve and sleeveless approach

**Fig. 6:**
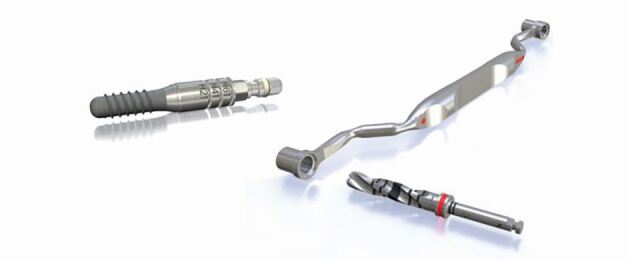
Straumann drill spoons. Image reproduced with permission from Straumann Group UK

**Fig. 7:**
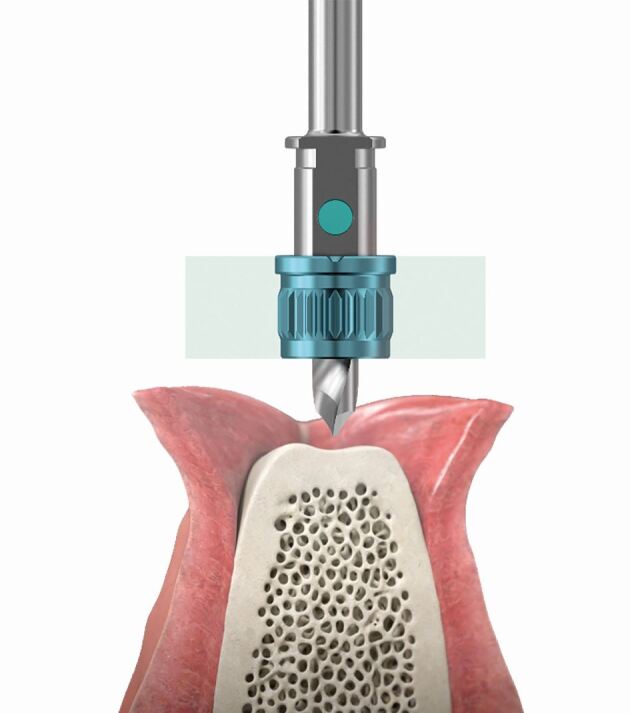
Anthogyr drill guide sleeves. Image reproduced with permission from Straumann Group UK

**Fig. 8:**
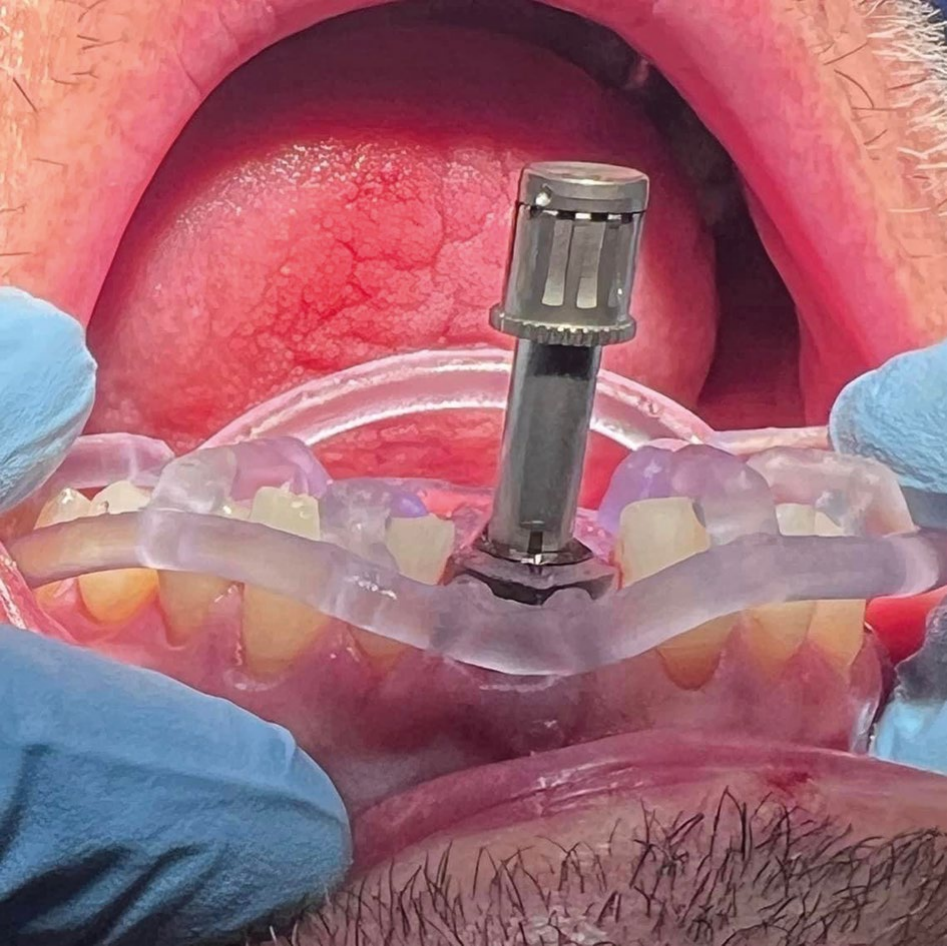
Anthogyr drill guide sleeves and an example in use

Different implant manufacturers utilise ‘spoons', ‘keys', or cylinder ‘sleeves' to sit within the drill guide template. This is so that there is a barrier between the drills and the plastic the drill guide is made from. For example, the drill kit from Straumann uses exchangeable ‘keys' which sit into the guide. Increasing lengths of drills are then used, which take the osteotomy to full length before the key is exchanged and the osteotomy widened (Fig. 6, Fig. 7, Fig. 8). The drills themselves have height stops which prevent the drills and the implant guide mount from going deeper than that planned.

### Sleeves or sleeveless approach

Prosthetically guided implant placement has revolutionised dental implantology by offering enhanced precision and predictability. Within this technique, the use of a metal sleeve as a drilling guide has been widely adopted. However, it is vital to understand the scientific reasoning behind how the utilisation of metal sleeves impacts the accuracy of implant placement compared to sleeveless drill approaches. This discussion will summarise the results and conclusions from relevant literature and provide insight into the underlying mechanisms influencing accuracy.

Studies investigating the impact of metal sleeves on implant placement accuracy have consistently demonstrated improved precision. El Kholy *et al*.^30^ conducted a study comparing the accuracy of guided implant placement using metal sleeves versus sleeveless drills. They found that the use of metal sleeves significantly reduced deviations in implant angulation and position. The metal sleeve acted as a stabilising guide during drilling, minimising the potential for errors and enhancing overall accuracy.

These findings align with a systematic review conducted by Van Steenberghe *et al*.^31^ which encompassed various studies exploring the accuracy of computer-guided implant placement with metal sleeves. The review concluded that the use of metal sleeves led to superior accuracy in terms of implant position and alignment with the desired prosthetic outcome. The metal sleeve provided guidance and stability during drilling, reducing the likelihood of deviations.

**Fig. 9:**
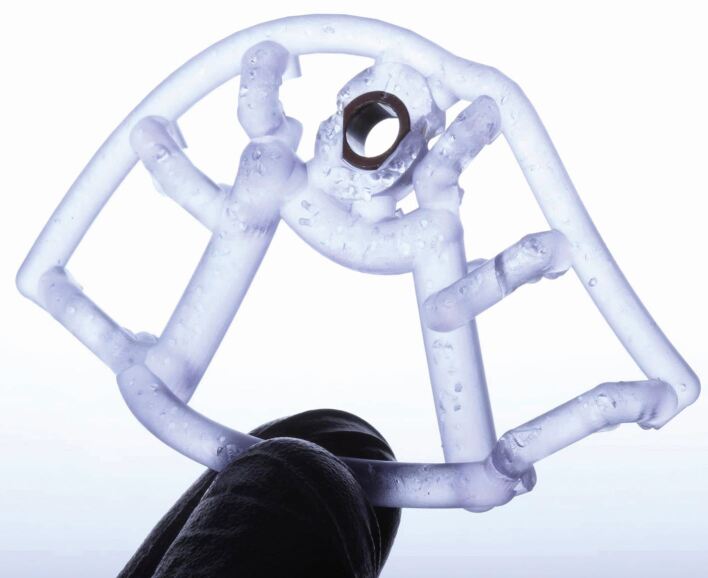
A 3D-printed SMOP implant guide with Anthogyr sleeve

The scientific reasoning behind the improved accuracy with metal sleeves can be attributed to several factors. First, the metal sleeve acts as a guide, ensuring precise alignment of the drill with the planned implant position. It eliminates the potential for manual errors that may arise when using sleeveless drill approaches, where the operator relies solely on visual estimation or hand-eye coordination. The rigid nature of the metal sleeve minimises deviations caused by unintentional hand movements, leading to enhanced accuracy (Fig. 9).

Furthermore, the metal sleeve offers stability during the drilling process. It prevents lateral movement of the drill, reducing the risk of deflection or deviation. The precise fit between the sleeve and the drill restricts any wobbling, ensuring that the drilling occurs along the intended trajectory. This stability contributes to maintaining the planned angulation and position, thereby improving the overall accuracy of implant placement.

**Fig. 10:**
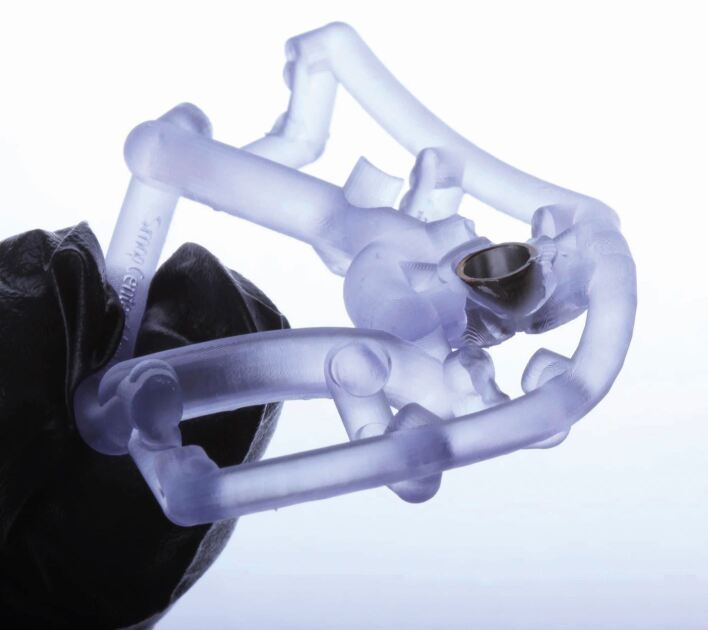
An underside view of a 3D-printed SMOP implant guide with Anthogyr sleeve showing how the STL cut of the sleeve hub has been restored through the use of a sleeve

However, it is essential to consider other factors that may influence accuracy when utilising metal sleeves. Oh *et al*.^32^ conducted a study investigating the impact of drilling depth, sleeve design and bone density on implant placement accuracy with metal sleeves. They highlighted the significance of selecting an appropriate drilling depth, as inadequate depth may lead to incomplete seating of the sleeve, potentially compromising accuracy (Fig. 10).

Additionally, the design of the sleeve, such as the presence of flutes or vents, can affect stability and accuracy. Finally, bone density plays a crucial role, as variations in density can impact the stability of the metal sleeve during drilling.

While metal sleeves have shown significant advantages in accuracy, studies exploring sleeveless drill approaches have presented comparable results. Lee *et al*.^33^ conducted a study comparing implant placement accuracy using a sleeveless drill technique with guided surgery using metal sleeves. The results demonstrated similar levels of accuracy between the two approaches. This suggests that sleeveless drill approaches can be a viable alternative, especially in cases where the operator possesses a high level of proficiency and dexterity in achieving precise implant placement.

However, sleeves also have the added benefit of a predictable control in all guides versus the sleeveless approach, which may not be fully encapsulated to the full depth of the sleeve in the production guide. With sleeveless approaches, the STL impression determines the guide sleeve shape as the fitting surface is cut from this.

There isn't this problem with metal sleeves. You can still see that the sleeve is cut away, but having the metal sleeve present restores the control and hold around the drill.

In conclusion, the use of metal sleeves in prosthetically guided implant placement has consistently demonstrated improved accuracy compared to sleeveless drill approaches. The scientific reasoning behind this improvement lies in the metal sleeve's ability to provide guidance and stability during drilling, reducing deviations in angulation and position. However, factors such as drilling depth, sleeve design and bone density should be carefully considered to optimise accuracy when utilising metal sleeves, while sleeveless drill approaches have shown comparable.

## Guided surgery drilling and implant placement

### Type of guided implant surgery drill and placement protocol

**Fig. 11:**
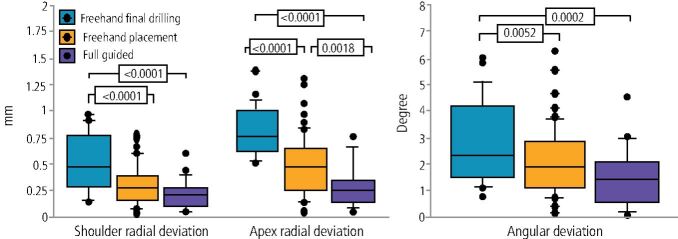
Investigated error from guided surgery with various implementations. Image reproduced with permission from Behneke *et al.*, ‘Factors influencing transfer accuracy of cone beam CT-derived template-based implant placement', *Clinical Oral Implants Research*, 2011, Wiley^13^

Once the data matching has been completed, the next step in the guided implant placement procedure is using the drills with the drill guide that has been created. The methodology of guided surgery varies depending on how ‘guided' the process is. We can categorise the variations of drilling protocol into three varieties:

1. Free-hand final drilling - the initial drill is the only part of the drilling sequence of widening the osteotomy that is guided by the template. Widening and/or lengthening the initial pilot hole is then done manually
2. Free-hand placement - all drilling sequences are guided, but the placement of the implant into the osteotomy created is free-hand after the removal of the surgical guide
3. Fully guided drill sequence and placement - this is the most recent evolution of guided surgery, where every step of both the creation of the osteotomy and the placement of the implant using a guide mount is done through the surgical guide (Fig. 11).

**Fig. 12:**
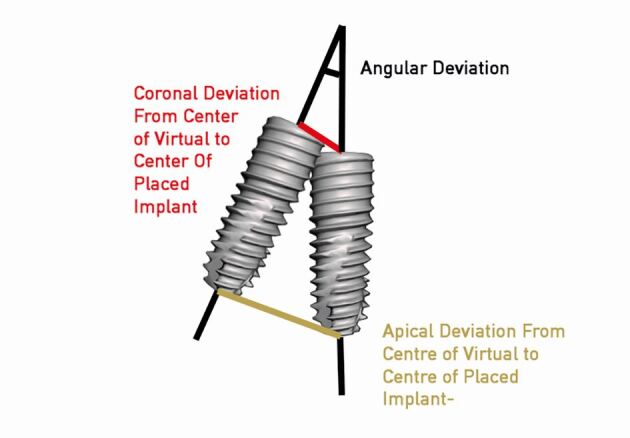
The positions used as reference points for the coronal, apical and angular deviations

The accuracy of this step would factor into how accurate the overall procedure is. Behneke *et al*.^13^ investigated the factors that would influence the drilling protocol. The study measured the final placement against the virtual in three respects:

- Coronal radial deviation
- Apex radial deviation
- Angular deviation (Fig. 12).

The study was a comparatively large in vivo study with 52 partially edentulous patients having a total of 132 implants placed. Of the 132 implants placed, half were placed with an open surgical flap and half were placed flapless. A total of 86 of the implants were placed completely free-hand, 24 guided only with the pilot drill, and 22 with fully guided drilling and placement protocols.

The results showed that the fully guided drill sequence and implant placement were significantly more accurate than the less fully guided approach, which in turn was more accurate than the free-hand final drilling and placement approach.

The effect of pilot guided versus fully guided was looked at in two RCT studies which reviewed the accuracy of guided surgery using each protocol.^34^^,^^35^There were statistically significant differences found for each accuracy parameter, with fully guided providing the most accuracy.

### Accuracy of flap versus flapless surgical technique

**Fig. 13:**
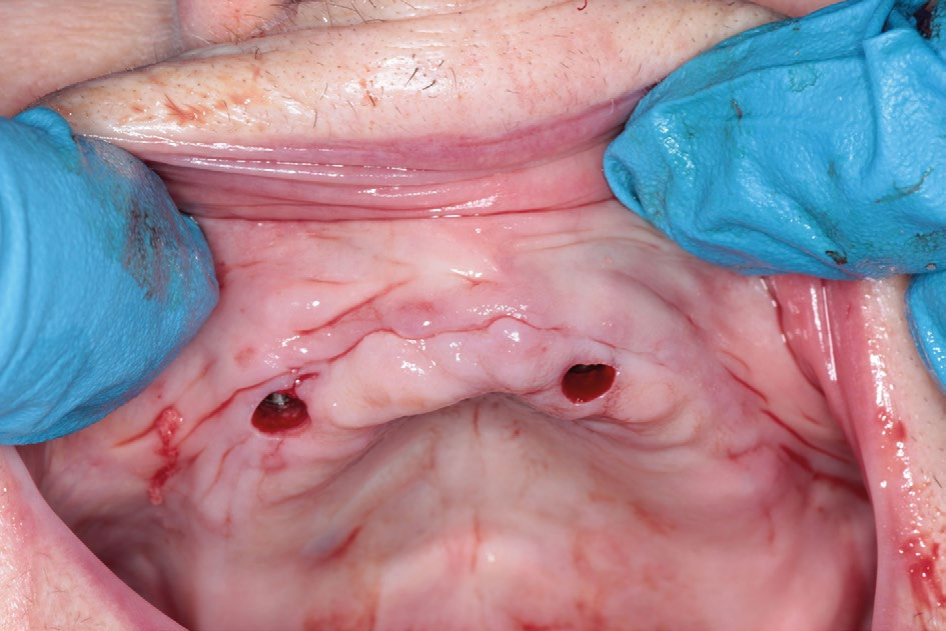
Flapless guided implant surgery in an edentulous arch

**Fig. 14:**
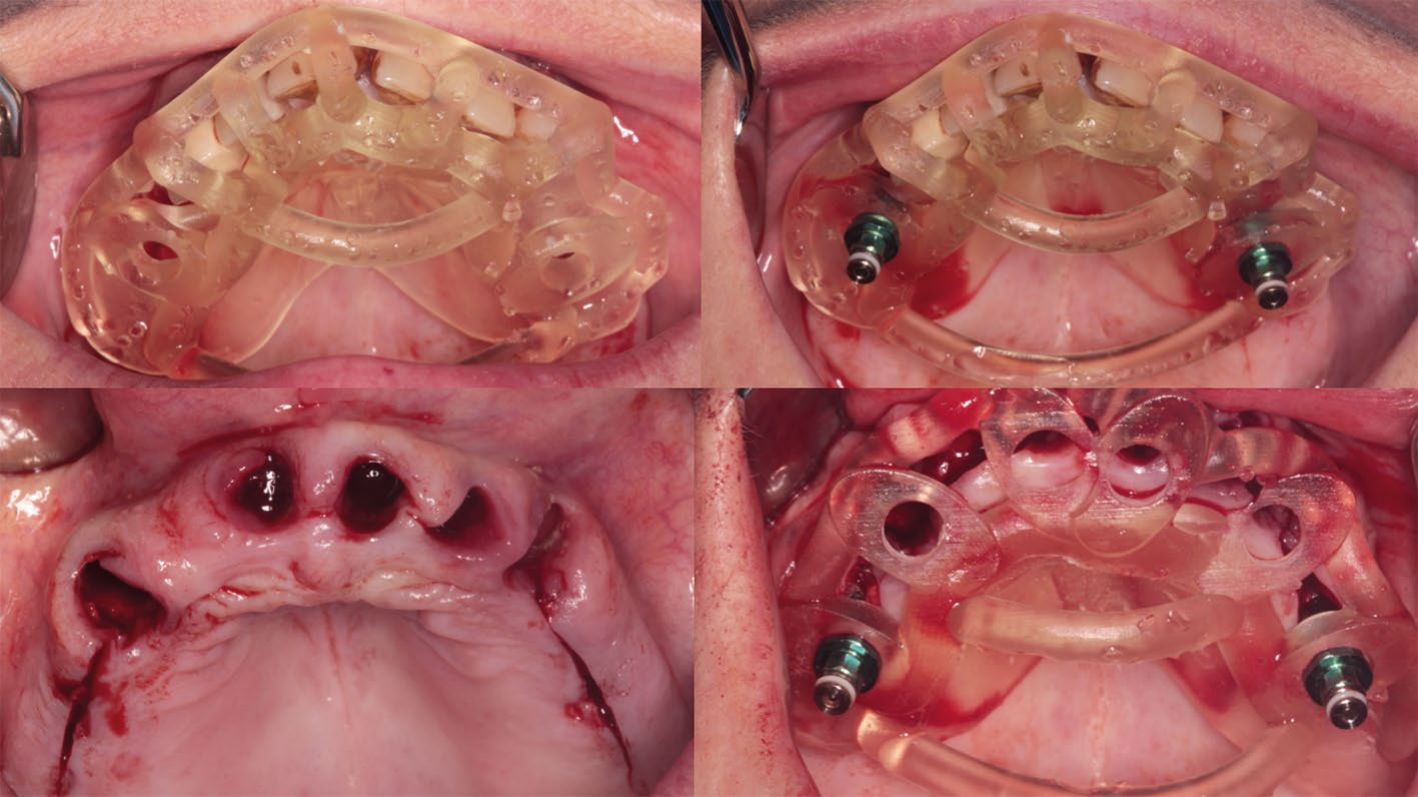
Two-staged dentate, then edentulous guided implant surgery

**Fig. 15:**
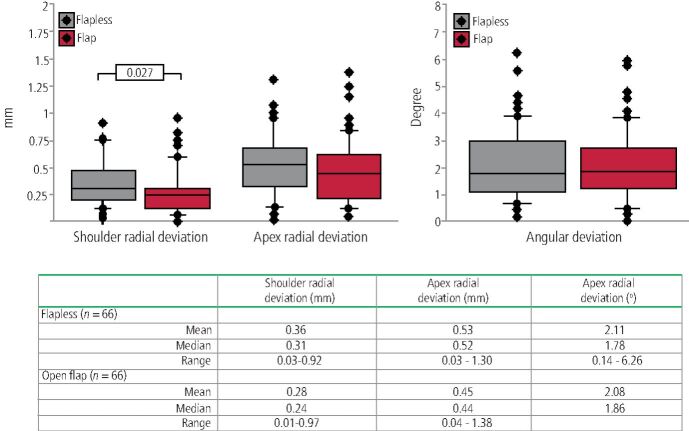
Investigated error from guided surgery, flapless and with flap. Image reproduced with permission from Behneke *et al.*, ‘Factors influencing transfer accuracy of cone beam CT-derived template-based implant placement', *Clinical Oral Implants Research*, 2011, Wiley^13^

Behneke *et al*.^13^also studied the accuracy of various types of guides, including whether a flap was introduced into the guided surgery procedure, and stated in the conclusion that ‘flap elevation did not negatively influence the positioning of the tooth-supported surgical templates and that the natural dentition allowed a sufficient anchorage', based on the results showing no statistically significant differences in the linear deviation at the apex while there was a small borderline statistically significant (p = 0.027) effect on the difference in coronal radial deviations (Fig. 13, Fig. 14, Fig. 15).

Behneke's^13^ study correlates with other studies^25^^,^^36^^,^^37^which also did not find a difference in accuracy. Flapless surgery can therefore be said to not introduce new levels of errors, while raising a surgical flap allows any errors that do occur to be seen.

## Human error

Komuro also found that numerous factors, including technology, software and human error, may cause CBCT findings to deviate from their true values.^28^ Edge enhancement and metal artefact reduction procedures increase the amount of software correction required. Regarding human error, it is generally agreed that the person who is taking the measurements is the most at fault, as the user may find it difficult to see a precise margin in a CBCT scan. Machine learning and AI data matching can help this process, where average values can be used to create a more accurate overall match. Up-to-date software is therefore paramount.^38^

Another factor that can impact placement that has not yet been discussed in this thesis is the variation in surgical guide drill systems among different manufacturers. Depth control is possible through specific steps in some systems but not in others (which use indication lines instead). In some systems, a pilot preparation is made, whereas in others, the full osteotomy is prepared through the guide. Finally, some systems also provide a mechanism for placing the implant itself through the guide. There is also the limitation of the drill and component tolerance which causes wobble, leading to deviations through mechanical means. These deviations would be increased further from the point of wobble; therefore, the guide-to-bone distance would influence deviation. These various components may lead to errors in placement in the coronal position, apical position and angulation (Fig. 12).^39^

A study by Sarment *et al*.^40^ assessed these deviations by comparing the effects of different drill systems. An angular deviation of 3.81 with a high of 24.9 was observed across all systems in general. Although these discrepancies seem to be significant, there is no in vivo RCT that compares computer-guided versus conventional surgery (with or without the use of any form of a surgical template) to support a claim of guided implant surgery being more or less accurate. Several minor in vitro investigations compared surgical deviations for conventional analogue surgery with surgical deviations for computer-guided surgery. In all cases of deviation, a statistically significant improvement was shown in favour of guided surgery. When the angular deviations were compared, for example, they were 4.5° for guided and 8.0°, 4.2° and 10.4°, respectively.^40^

Several other important factors, such as the following, may also have an impact on overall accuracy:^24^^,^^41^^,^^42^

- Determination of bone volume in CBCT viewing. The accuracy and precision with which a clinician can determine the precise position of a thin piece of tooth or scanning appliance in a CBCT scan
- The reliability of the 3D intra-oral scan
- The accuracy of the data merging process
- The reliability of the 3D-printed surgical template
- Surgical guide movement and fit during the clinical placement of the implant.

It is critical to understand the accuracy of each of the steps to form a full conclusion on the overall accuracy of the novel method to combine datasets and perform minimally invasive edentulous guided surgery.

## Discussion

### Overall accuracy of guided implant placement

Van Assche *et al*.^39^ carried out a retrospective study into the accuracy of guided implant placement as measured across various clinical studies: ‘meta-analysis revealed a mean error of 0.99 mm (ranging from 0-6.5 mm) at the entry point and of 1.24 mm (ranging from 0-6.9 mm) at the apex. The mean angular deviation was 3.81° (ranging from 0-24.9°). Significant differences for all deviation parameters were found for implant-guided placement compared to placement without guidance. The number of templates used was significant, influencing the apical and angular deviation in favour of the single template. Study design and jaw location had no significant effect'.

Schneider *et al*.^43^ also conducted a systematic review, isolating eight articles out of 3,120 titles regarding the accuracy of guided implant surgery and ten regarding clinical performance. The authors conducted a meta-regression analysis across the literature gathered and found that the mean deviation at the entry point was 1.07 mm (95% CI: 0.76-1.22 mm) and the mean apex deviation was 1.63 mm (95% CI: 1.26-2 mm). However, the studies included in this meta-analysis were based on different technologies and on both artificial bone blocks and in the mouth, with meta-analysis considered studies before 2009. Therefore, the meta-analysis is difficult to compare to current guided surgery methods as both software and guide drill systems have materially progressed since these studies were undertaken.

One point to consider is that for most historical studies evaluating the accuracy of implant placement, radiographic methods have been used where pre- and post-operative CBCT images are taken. More recent studies have used alternative, non-radiographic means using model scanning with a lab light scanner after impressions are taken or have used intra-oral scan data to determine the implant position.^44^^,^^45^^,^^46^ Pyo *et al*. have reviewed and quantified the differences between these, comparing and describing the techniques used.^47^

Other studies have shown no significant difference between the radiographic and non-radiographic methods, which is obviously better for patient dose-related concerns.^48^^,^^49^^,^^50^

### Factors influencing both dental implant success from the application of digital planning

Digital planning can have an impact on the success of implant placement in terms of the following factors.

#### Bone resorption

Gingival height and width to maintain papilla and prevent recession for long-term soft tissue stability. Teughels *et al*.^51^ conducted a systematic review of articles on Pubmed, Cochrane and the ISI databases to identify eligible human studies discussing the aesthetic outcomes of implants to give a reflection into the critical dimensions required around implants for optimal aesthetic outcomes. The review concluded that the optimal distance between tooth and implant, based on various literature and studies, was 3-4 mm based on one cross-sectional and two prospective case series (which 75-87% of the time led to complete papillary infill).

#### Implant bucco-palatal position

An article from Evans *et al*.^52^ where a retrospective review was carried out analysed the aesthetic outcomes of 42 non-adjacent implant restorations and after function related their bucco-palatal position to the amount of gingival recession that occurred. The paper concluded that implants with a buccal coronal position (1.8 mm + -0.83) showed nearly three times more recession than implants that were positioned more palatally (0.6 mm + -0.55 mm). However, the study involved teeth that had a mean function time of 18.9 months, but the results were found to be highly statistically significant (p = 0.000).

Another more extensive study by Spray^53^ studied the amount of buccal bone recession or gained on the placement position of over 3,000 implants. The study concluded that for implants placed with more than 1.8-2 mm of buccal facial bone present on placement, the vertical height of bone on the facial aspect of the implant either increased or bone loss was significantly reduced compared to those implants with less than 1.8 mm present on placement.

#### Implant positioning effects on soft tissue stability

Nowhere is the effect on soft tissue stability more critical than in the aesthetic zone, that is, around the exposed anterior teeth. Soft tissue recession can play a significant role in whether the restoration placed is deemed a success or a failure.

When a tooth is extracted, the bone resorbs, and the soft tissue therefore is unsupported.

Computer-guided implant placement has the potential to be considerably more accurate than unguided placement, but there are risks associated with it.^39^ By using the digital plan created during the virtual planning procedure and then sending the 3D position from the pre-surgical planning to the dental laboratory, it is possible through CAD/CAM to manufacture a pre-fabricated fixed prosthesis that can be attached directly to newly placed implanted fixtures. In an edentulous case, this is a significant advantage to the patient, where the period of edentulism can be minimised during the healing period.^31^ However, for this pre-planned prosthesis to fit passively, the placement of the implant through the drill guide must be accurate to follow the virtual plan created.^3^ If inaccuracy results in a deviation from this plan, the variations in fit may mean that this prosthesis either does not fit or does not fit passively.^20^

## Conclusion

In conclusion, this literature review explores prosthetically designed implant placement and its influence on dental implant success. By examining the factors that impact implant outcomes, this review aims to provide clinicians with a comprehensive understanding of the key considerations and best practices in achieving successful implant placements. Ultimately, this knowledge can contribute to enhancing patient care and the long-term success of dental implant treatments.
